# A natural language processing approach to determine whether streamlined health technology assessment for multi-indication medicines is feasible

**DOI:** 10.1371/journal.pdig.0001657

**Published:** 2026-08-26

**Authors:** Isaiah Luc, Drew Carter, Erik Bergman, Gabriel Westman, Tracy Merlin

**Affiliations:** 1 Adelaide Health Technology Assessment, Adelaide University, Adelaide, South Australia, Australia; 2 Swedish Medical Products Agency, Uppsala, Sweden; 3 Department of Medical Sciences, Uppsala University, Uppsala, Sweden; Yonsei University College of Medicine, KOREA, REPUBLIC OF

## Abstract

Conducting a health technology assessment (HTA) for each indication of a medicine contributes to increasing demand on HTA systems and results in delays in access. Streamlining assessments for subsequent indications has been proposed, but there is limited evidence on how this could be achieved. We aimed to quantify semantic similarity between HTA documents for different indications of the same medicine to explore whether concepts could be carried forward to subsequent assessments. Public Summary Documents (PSDs) from July 2019 to November 2024 were included if they related to new major submissions to the Australian Pharmaceutical Benefits Advisory Committee. Each paragraph was embedded using the Sentence-Bidirectional Encoder Representations from Transformers (SBERT) model. Pairwise Euclidean distances (measuring semantic similarity) were quantified between embeddings across indications for multi-indication medicines and were compared with those for single-indication medicines. Cluster analysis, followed by manual thematic review, identified recurrent concepts across indications of the same medicine. A total of 326 PSDs were included (154 single-indication medicines, 59 multi-indication medicines). Multi-indication medicines demonstrated significantly greater semantic similarity across their indications compared with single-indication medicines. However, recurrence of specific concepts across either all or similar indications of a medicine was infrequent. Despite the greater semantic similarity observed across indications of multi-indication medicines, this did not translate into substantial opportunities to carry forward assessment concepts. Therefore, it is unlikely that any concepts or information from assessments undertaken for previous indications could be carried forward to subsequent indications. The methods described provide a foundation for future research on semantic overlap in HTA and other policy areas.

## Author summary

When a new medicine is evaluated for public funding, it is usually assessed separately for each medical condition it treats. As medicines are increasingly approved for multiple conditions, this approach places growing pressure on health technology assessment systems and can delay patient access. Some have suggested that parts of earlier evaluations could be reused when the same medicine is assessed for a new condition. However, there has been little evidence to show whether this is feasible. This study uses machine learning-based analysis to examine whether assessment documents for the same medicine, but for different conditions, contain recurring concepts that could realistically be carried forward. We analysed more than 300 reimbursement decisions in Australia. Although assessments of the same medicine were more similar to each other than they were to assessments of unrelated medicines, we found that evaluation concepts rarely repeated consistently across conditions. This indicates that simply reusing content from earlier assessments may not be appropriate in most cases. Beyond this specific application, our approach demonstrates how machine learning can be used to measure similarity across other types of health policy documents. Such methods could help policymakers determine other processes that can be streamlined, such as assessments across different jurisdictions.

## Background

Medicines with multiple indications (multi-indication medicines) pose a significant challenge to the efficiency of health technology assessment (HTA) processes [1]. In most jurisdictions that practice HTA to inform the reimbursement of medicines, the assessment is done on a per-indication basis [1]. As a result, pharmaceutical companies are required to submit separate applications for each indication of a medicine, which is then followed by months of assessment work by HTA and decision-making agencies [1,2]. This approach contributes to the increasing demand on HTA systems, many of which are already operating beyond capacity and results in delays to the subsidised access of new therapies for patients. In Australia, for example, the Pharmaceutical Benefits Advisory Committee (PBAC) has already had to delay reimbursement decisions as the number of applications exceeded the country's evaluation capacity [3]. Globally, the need for accelerated patient access to new medicines is also recognised [4]. In relation to multi-indication medicines, academic and industry stakeholders are asserting that repeated assessments of the same medicine (albeit for different indications) are creating significant delays for patients with different health conditions, leading to inequities in access [2,5,6].

Our recent scoping review identified that conducting a full HTA for the first indication, followed by streamlined assessments for subsequent indications, may be a promising strategy to improve the upfront efficiency of HTA for multi-indication medicines [1]. Streamlined assessments could be supported by the use of subsequent real-world evidence (RWE) or confirmatory trial data once available. Medicines Australia, the Australian pharmaceutical industry body, highlighted this approach when the PBAC was reviewing subsidy options for programmed death-(ligand) 1 (PD-(L)1) medicines in 2018 [5].

However, any streamlined assessment must adequately preserve rigour and transparency to ensure that the HTA process retains legitimacy and that public resources are only allocated to uses that provide sufficient value. To our knowledge, no HTA agency has implemented streamlined assessment for multi-indication medicines, potentially due to the absence of research into how HTA methods should be streamlined, i.e., simplified or narrowed [1]. A recent study argues that, in some jurisdictions, pricing agreements for multi-indication medicines (e.g., multi-year multi-indication agreements) did not lead to a substantial decrease in wait times for patients, because the full HTA is still conducted [6]. This highlights the significance of a streamlined HTA for subsequent indications to accelerate patient access.

We have previously hypothesised that conducting a full HTA for each subsequent indication of a multi-indication medicine may result in duplication of effort, for example, through repeated evaluation of the same safety evidence across indications (as the same molecule should have the same safety profile). Medicines Australia suggests that streamlining assessment for subsequent indications may be reasonable on the basis that HTA evaluators and the PBAC are already familiar with the molecule [5]. Evidence of duplication of effort (one notable form of inefficiency) would likely manifest as overlap in text content and meaning (semantic overlap) across HTA reports for different indications for the same medicine, i.e., there would be repetition in meaning as similar things are being written, e.g., about the safety evidence of a molecule. Therefore, a promising starting point for streamlining assessment is to identify semantic overlap (text that has the same meaning) across evaluations of the same medicine. This could guide efforts to reduce the scope for duplication of effort, e.g., by carrying forward molecular descriptions or safety evaluations of the medicine for subsequent indications. Accordingly, we sought to identify the extent of semantic overlap between HTA reports for different indications of the same medicine, namely as a means of identifying where and how an assessment could be streamlined. Specifically, the research questions for this study were:

1. Is there greater semantic similarity between HTA reports for different clinical indications of the same medicine than between HTA reports of different medicines which have only one indication?
2. How frequently and where do semantically overlapping concepts recur across HTA reports for indications of the same medicine?

For the purposes of this study, semantic similarity refers to the degree or magnitude of similarity in meaning whereas semantic overlap refers to two text chunks conveying the same meaning.

## Methods

### Natural Language Processing (NLP) approach

Initially a manual, narrative approach was explored for identifying similarities across Public Summary Documents (PSDs). These documents summarise the information in HTA reports and reimbursement outcomes for medicines submitted for funding under Australia's Pharmaceutical Benefits Scheme (PBS) [7]. However, this approach was labour intensive and would have required a sampling framework if manual extraction was to be undertaken because the volume of potential PSDs was too large. Given this limitation, and because we wanted to include all available information, we adopted a validated Natural Language Processing (NLP) approach to identify semantic similarity between PSDs.

We have previously made extensive use of the Sentence-Bidirectional Encoder Representations from Transformers (SBERT) model for similarity and clustering analyses in medicine regulatory documents [8–11]. For example, previously we have used SBERT to explore the semantic alignment of European Public Assessment Reports (EPARs) and European Medicines Agency (EMA) guidelines and to identify how textual uncertainty is communicated across EPARs [8,10]. Building on this experience, we developed and implemented an NLP approach to identify semantic similarity and overlap across PSDs.

### Data acquisition and pre-processing

Medicine submissions to the PBAC between July 2019 and November 2024 were screened on the PBS website [7]. Submissions were included if they met our inclusion criteria of being a Category 1, 2 or major submission, considered at a major PBAC meeting (March, July or November) and had a corresponding published PSD. Submissions were excluded if they: did not relate to a new PBS listing; simply amended an existing listing; were a resubmission; or were submitted by the Committee Secretariat (i.e., were not an industry submission). These criteria were selected to focus on assessments which require the most amount of evaluation effort.

PSDs are not the full HTA report (these are not available due to commercial-in-confidence agreements), they do, however, summarise the content of the evaluation of a submission, and the rationale for the Committee’s funding decision. While they are named as “summary documents”, they are often quite extensive and detailed, in some cases exceeding 70 pages, reproduce the executive summary of the full report and report the key decision-making considerations underpinning PBAC recommendations. Thus, we considered this to be the most appropriate source to examine semantic similarity and overlap across indications of a medicine.

Documents were then automatically sectioned into the following: population and disease, comparator, clinical evidence of effectiveness, safety, economic analysis, financial implications, PBAC outcome and the full document, according to the headings used in the document. Each section was further segmented into chunks at the paragraph level as typically each paragraph conveys a distinct semantic concept. Text chunks were encoded into semantic text embeddings using the SBERT model (*all-mpnet-base-v2).* Additional technical detail is provided in S1 File.

### Euclidean distance analysis

We quantified semantic similarity by calculating the Euclidean distance between embeddings, where lower values indicate more similar meaning [8,12]. Multi-indication medicines (i.e., medicines with more than one PBAC submission included in our analysis) were grouped separately from single-indication medicines. All analyses were conducted separately for each predefined section of the PSD as well as for the full document.

For single-indication medicines, pairwise distances were calculated between all text chunks across PSDs. Document-level similarity was summarised using the mean of the closest-matching text segments. This method captured the strongest semantic overlap between documents while minimising the influence of non-matching or unrelated content and was adapted from our previous full document semantic matching analysis [8]. More detail on this method can be found in S1 File.

For multi-indication medicines, the pairwise Euclidean distances were calculated between every possible indication pair within each multi-indication medicine (e.g., first indication vs second indication, first indication vs third indication, second indication vs third indication, etc.). That is, the same approach as the single-indication medicine group was used but on a per-medicine basis.

#### Statistical analysis.

As described above, we derived a document-level Euclidean distance for each PSD pair. For the single-indication medicine group, one triangle of the pairwise similarity matrix formed the set of observations for analysis (as the matrix was symmetrical). For multi-indication medicines, a separate similarity matrix was generated for each medicine, because each medicine had multiple PSDs (i.e., multiple indications) that could be compared pairwise. One triangle of each matrix was then averaged to yield a single similarity value per medicine. These medicine-level observations were compared between groups using an independent samples t-test.

#### Sensitivity analysis.

We performed a bootstrap sensitivity analysis to test whether the observed differences between single and multi-indication medicines were influenced by the averaging framework applied (one global average for single-indication medicines versus the two-step averaging process for multi-indication medicines). More detail is provided in S1 File. We compared the baseline single indication group mean to the bootstrap distribution by calculating the empirical p-value and 95% percentile intervals.

### Cluster analysis

We applied a cluster analysis approach to identify groups of text chunks with a high degree of similarity or semantic overlap across all PSDs, consistent with our previous work on medicinal regulatory documents [9,10]. The Density-Based Spatial Clustering of Applications with Noise (DBSCAN) algorithm (with Euclidean distance as the metric) was run on all embeddings from text chunks in the single and multi-indication medicine groups [13].

This cluster analysis was used to address the second research question by identifying how frequently specific evaluation concepts recurred across HTA reports for indications of the same medicine. This differed from the Euclidean distance analysis (above), which quantified the degree of semantic similarity between PSDs and their predefined sections.

#### Parameter selection.

There are two DBSCAN parameters that need to be tuned for the dataset: (1) epsilon (ε), which is the maximum Euclidean distance between two text chunks for them to be considered a cluster (i.e., the sensitivity) and (2) minimum samples, namely the smallest number of text chunks that a cluster can have [13]. Minimum samples was set to 2 to identify semantic overlap in medicines with just two indications. Parameter selection for ε followed standard practice, inspecting the distance distributions, evaluation of clustering quality and visualising the clusters in the embedding space [10]. Selection of the final parameter was based on manual inspection of cluster members to confirm semantic consistency in the resulting clusters, i.e., reading text chunks and confirming semantic overlap. A more detailed explanation is provided in S1 File.

#### Cluster filtering and labelling.

After DBSCAN, we applied two deterministic filters to identify semantic clusters which aligned with our research aim (i.e., identifying semantic overlap across indications of the same medicine). In Filter 1 (F1), we only included clusters which had a member (i.e., a text chunk) from every indication of a multi-indication medicine. In Filter 2 (F2), we grouped indications within a multi-indication medicine based on the Australian Refined – Diagnosis Related Group (AR-DRG) version 11 codes with the intention of similar indications being grouped together [14]. Ignoring indication groups of 1, clusters were retained if they contained at least 1 member from every indication of an indication group. This was chosen as we wanted to investigate semantic overlap spanning indications of a medicine in the same disease area (as opposed to all indications). Only cluster members that contributed to the filtering predicates (in F1 and F2) were considered. Clusters that had members which were not semantically aligned were labelled as noise and excluded. Similarly, individual cluster members that were not semantically aligned with the rest of the cluster, were labelled as noise and excluded. When a cluster clearly contained multiple distinct themes or different medicines, it was split into separate clusters, and these clusters were re-evaluated and retained only if they met the filtering criteria.

*Python 3.13.3* was used for all text processing and data handling, with libraries NumPy, pandas, scikit-learn (DBSCAN, PCA, t-SNE, NearestNeighbors, and clustering metrics), sentence-transformers (SBERT embeddings), SciPy (stats), Matplotlib and Seaborn for visualisation.

**Fig 1:**
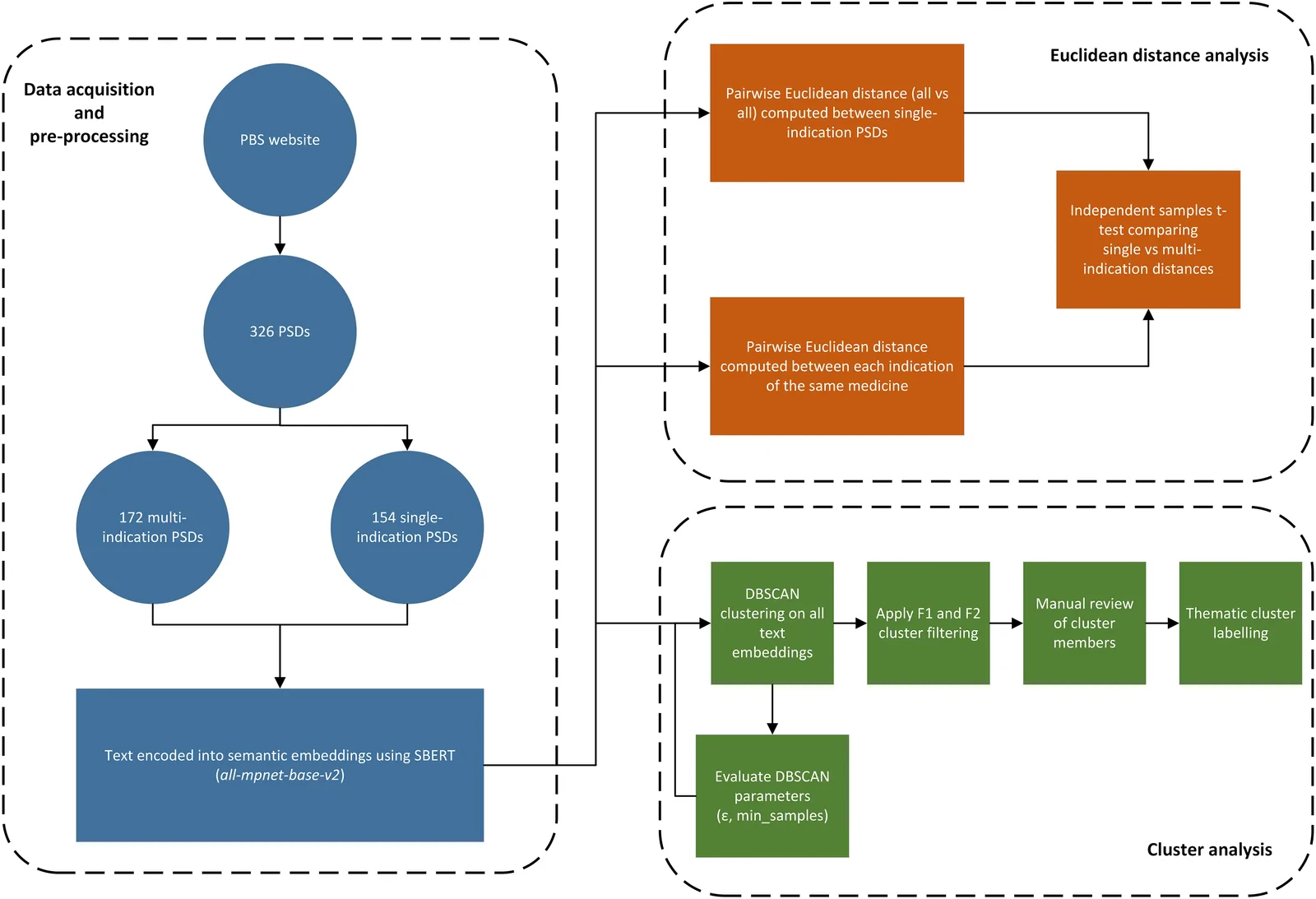
Overview of the NLP workflow. Abbreviations: DBSCAN; Density-Based Spatial Clustering of Applications with Noise, NLP; natural language processing, PBS; Pharmaceutical Benefits Scheme, PSD; Public Summary Document, SBERT; Sentence-Bidirectional Encoder Representations from Transformers.

An overview of the NLP workflow is presented in Fig 1 and a more detailed discussion of the NLP approach is provided in S1 File.

## Results

### Data acquisition

Dating from July 2019 to November 2024, 326 PSDs met the inclusion criteria and were downloaded. From these PSDs, 154 single-indication medicines and 59 multi-indication medicines were identified. A total of 172 PSDs were related to multi-indication medicines.

### Euclidean distance analysis

**Fig 2:**
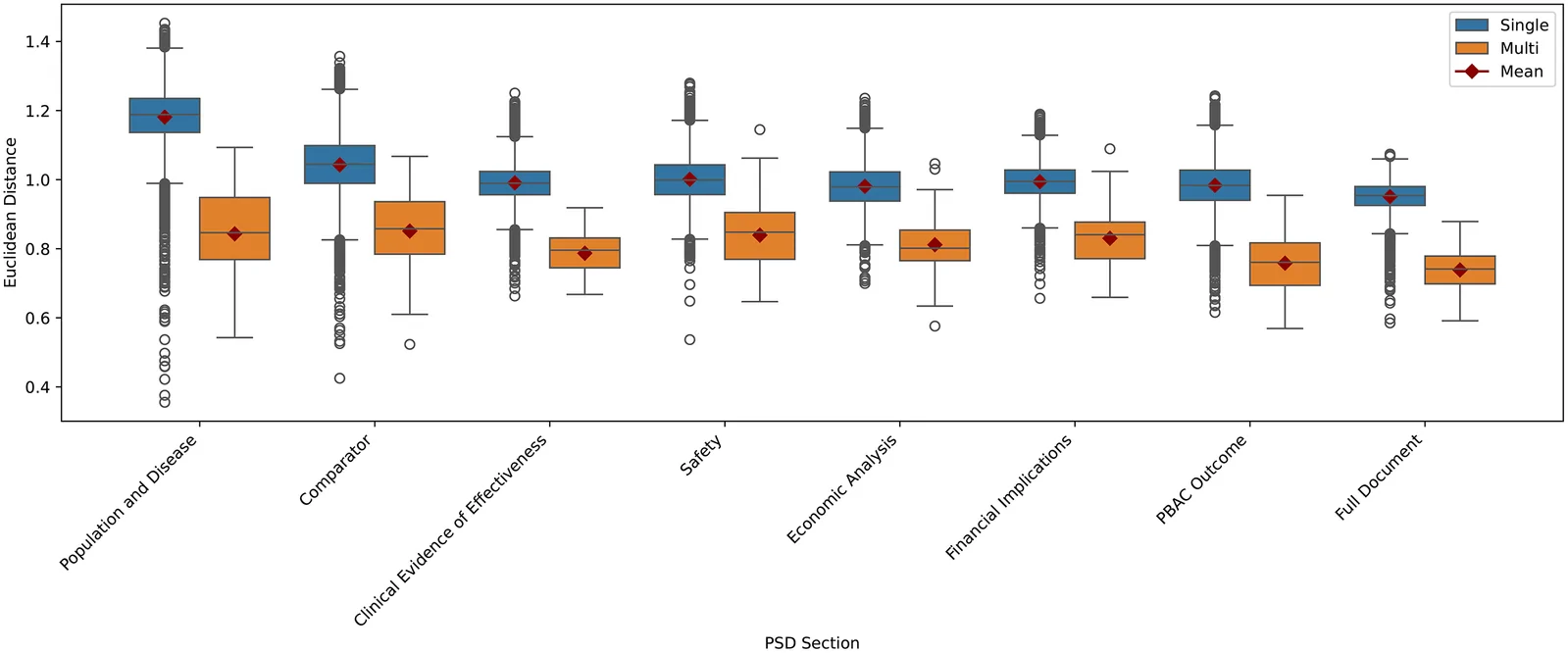
Box plot of Euclidean distances comparing single and multi-indication medicines across PSD sections. Note: lower Euclidean distances indicate greater semantic similarity. Diamonds represent group means. Abbreviations: PBAC; Pharmaceutical Benefits Advisory Committee, PSD; public summary document.

Fig 2 shows the distribution of Euclidean distances across all document sections for the single versus multi-indication medicine groups. Across every section, multi-indication medicines displayed lower distances than single-indication medicines, indicating greater semantic similarity. The greatest difference between the two groups was observed in the population and disease section, whereas the safety section had the smallest difference. Within multi-indication medicines, the clinical evidence section had the lowest absolute Euclidean distance. The full-document analysis also showed a clear separation, with multi-indication PSDs demonstrating greater overall semantic similarity than single-indication PSDs.

Formal statistical testing confirmed these visual patterns. Independent samples t-tests demonstrated that the mean Euclidean distances were significantly lower for multi-indication medicines across all sections and for the full document (all *p* < 0.001). Full results are presented in S1 File.

#### Sensitivity analyses.

Across all chunking approaches and sections, the baseline single indication medicine mean fell within the bootstrap 95% percentile intervals (summary results are presented in S1 File). These findings confirm that the choice of aggregation framework did not influence the results.

### Cluster analysis

#### Ε parameter selection.

Based on distance distributions, cluster quality metrics and visualisations and manual review of clusters, ε = 0.44 was selected as the value most appropriate for cluster labelling. Full details on parameter selection are provided in S1 File.

#### Cluster filtering.

Indication groups (based on AR-DRG groupings) for F2 filtering can be found in the S2 File. Seven multi-indication medicines were excluded as their indications did not belong to the same AR-DRG code. The remaining 52 medicines formed 57 unique medicine-AR-DRG groups. Broadly, oncology indications were grouped by tumour site, respiratory diseases were grouped together (e.g., asthma, chronic obstructive pulmonary disease), and immune-mediated diseases were grouped together (e.g. Crohn’s disease, ulcerative colitis).

Across 25,590 text chunks, DBSCAN assigned 4,682 (18%) text chunks (cluster members) to 1,718 clusters, while the remaining 20,908 (82%) were noise.

After F1 (clusters spanning all indications of a medicine) and F2 (clusters spanning similar indications of a medicine) filters were applied, 46 clusters (containing 196 cluster members) and 116 clusters (containing 358 cluster members) were retained for F1 and F2, respectively. After thematic review to enforce semantic alignment within a cluster, off-topic members were removed, heterogeneous clusters were reclassified as noise, and mixed-theme or medicine clusters were separated where necessary. See Table 1 for an example of how a cluster was reclassified as noise. The final numbers of included clusters were 49 clusters (containing 135 cluster members) and 123 clusters (containing 292 cluster members) for F1 and F2, respectively. The step-wise inclusion and exclusion workflow is shown in S1 File, and the final retained clusters and members are provided in the S2 File.

**Table 1:** Example of a cluster identified by DBSCAN which was reclassified as noise during manual review.

Medicine	Indication	Text	Reason for exclusion
Lorlatinib	Locally advanced (stage IIIB) or metastatic (stage IV) anaplastic lymphoma kinase-positive non-small cell lung cancer	The equi-effective doses proposed in the submission were lorlatinib 100 mg QD and alectinib 600 mg BID, based on the assumptions that the mean duration of treatment and the mean relative dose intensity in clinical practice would be the same for both drugs. The cost-minimisation approach taken in the submission was to equate the cost per day of lorlatinib with the cost per day of alectinib, assuming that the treatment duration with lorlatinib was going to be the same as alectinib. **The comparison of the point estimates for PFS suggested the treatment durations for lorlatinib and alectinib were likely to be similar thus the PBAC accepted the cost per day approach using the equi-effective dose of lorlatinib 100 mg per day and alectinib 1200 mg per day. The PBAC advised that the cost per day of lorlatinib should be no more than the cost per day of alectinib**	Although the language and methodological description were highly similar across both text chunks, the appraisal conclusions differed. In one submission, PBAC accepted the equi-effective dose approach, whereas in the other, PBAC raised concerns. As these texts did not convey the same meaning, they were excluded as a semantically overlapping cluster.
	Non-small cell lung cancer	The equi-effective doses were estimated as lorlatinib 100 mg daily and alectinib 600 mg twice daily, assuming the same mean duration of treatment for the two drugs. The equi-effective doses for lorlatinib and alectinib proposed in the submission represent the daily doses used in the CROWN and ALEX studies, respectively, and are the doses recommended in the corresponding Product Information for the two medicines. **There is uncertainty regarding the submission’s assumption that the treatment duration of lorlatinib will be the same as that for alectinib. Potential differences in PFS durations and toxicity/tolerability profiles between the two medicines may differentially affect their respective mean treatment durations.**	

Abbreviations: BID; twice a day, PFS; progression-free survival, PBAC; Pharmaceutical Benefits Advisory Committee, QD; once a day.

Notes: bolded text informed the reason for exclusion.

#### Cluster labels.

Each of the included cluster members (i.e., the text chunks) were assigned one of 22 labels which were created in an inductive and latent way after several rounds of data familiarisation. The cluster labels and their frequency of recurrence across clusters and medicines are presented in Table 2. As expected, F2 identified more clusters and involved more medicines than F1. For example, disease description increased coverage from 7 to 13 medicines and mechanism of action from 3 to 9 medicines. The top three most frequent labels were consistent across F1 and F2 filters: template text, consumer comments and disease description. However, under both filtering approaches these labels still had a low level of frequency with respect to medicine coverage (12% - 27% of multi-indication medicines).

**Table 2:** The frequency of cluster labels across clusters and medicines across the different filtering approaches.

Cluster label	F1		F2	
	Number of clusters	Number of medicines (of 59)	Number of clusters	Number of medicines (of 52)
Template text	12	11 (19%)	17	14 (27%)
Consumer comments	11	11 (19%)	15	12 (23%)
Disease description	7	7 (12%)	15	13 (25%)
Submission outcome	4	4 (7%)	8	8 (15%)
Table/figure notes	3	3 (5%)	10	7 (13%)
Mechanism of Action	3	3 (5%)	9	9 (17%)
Safety	2	2 (3%)	6	6 (12%)
Drug costs	2	2 (3%)	5	5 (10%)
Utilisation analysis details	1	1 (2%)	4	3 (6%)
Comparator	1	1 (2%)	3	2 (4%)
Listing details	1	1 (2%)	3	3 (6%)
Overview of the evidence	1	1 (2%)	1	1 (2%)
Economic model details	0	0 (0%)	10	3 (6%)
CMA details	0	0 (0%)	4	3 (6%)
Comparative effectiveness	0	0 (0%)	4	4 (8%)
RSA	0	0 (0%)	4	1 (2%)
Clinical trial details	0	0 (0%)	1	1 (2%)
QUM issues	0	0 (0%)	1	1 (2%)
Risk of bias	0	0 (0%)	1	1 (2%)
Subsequent therapy	0	0 (0%)	1	1 (2%)
Unmet clinical need	0	0 (0%)	1	1 (2%)

Abbreviations: CMA; cost-minimisation approach, QUM; quality use of medicines.

Notes: F1 (full-medicine coverage): keep clusters that include ≥1 text chunk from every indication of a multi-indication medicine. F2 (similar-indication coverage): within each medicine, keep clusters that include ≥1 text chunk from every indication in a group of similar indications (ignore groups of size 1, which reduces the number of eligible medicines to 52). Number of clusters does not equal the number of medicines as a single medicine can contribute multiple clusters within the same label.

A selection of clusters and their key concepts are presented in Table 3. For each cluster we report the cluster ID, medicine, two exemplar members drawn from different indications of the same medicine, and a brief “Key concepts” annotation summarising the shared themes captured by the cluster. These examples show (i) standardised text for the cost-minimisation approach (classified as template text), (ii) recurrent disease definitions and burden and prognosis language (classified as disease description), and (iii) consumer comments relating to the high need for listing and substantial clinical benefit (classified as consumer comments).

**Table 3:** Selection of text chunks illustrating semantic overlap across indications of medicines.

Cluster ID	Medicine	Indication	Text	Key concepts cluster addressed
**Cluster label: template text**				
2	bimekizumab	Severe active psoriatic arthritis	In the context of the cost-minimisation approach taken by the submission, a further consideration for PBAC is that, under Section 101(3B) of the National Health Act 1953, when the proposed medicine is substantially more costly than an alternative therapy, the committee cannot make a positive recommendation unless it is satisfied that, for some patients, the proposed medicine provides a significant improvement in efficacy and/or reduction of toxicity over the alternative therapy. If the committee is so satisfied, it must make a statement to this effect. The submission noted that PBAC had previously considered some treatments (UST, SEC, CZP, GUS, UPA and TOF) as ‘lower tier’ or less effective therapies and other treatments (ETN, ADA, IFX, IXE and GOL) as ‘higher tier’ or more effective therapies.	Standard template text for the CMA.
		Non-radiographic axial spondyloarthritis	In the context of the cost-minimisation approach taken by the submission, a further consideration for PBAC is that, under Section 101(3B) of the National Health Act 1953, when the proposed medicine is substantially more costly than an alternative therapy, the committee cannot make a positive recommendation unless it is satisfied that, for some patients, the proposed medicine provides a significant improvement in efficacy and/or reduction of toxicity over the alternative therapy. If the committee is so satisfied, it must make a statement to this effect.	
**Cluster label: disease description**				
1403	pembrolizumab	Early triple negative breast cancer	TNBC is defined by a lack of progesterone receptors (PgR) and oestrogen receptors (ER), as well as the absence of human epidermal growth factor receptor 2 (HER2) overexpression or amplification. According to an analysis of the Surveillance, Epidemiology, and End Results (SEER) database in the US, the five-year survival rate for someone with localised triple-negative breast cancer, is 91 percent. For cancer that has spread into nearby lymph nodes or nearby areas, the five-year survival rate is 65 percent. For cancer that has spread further into the body, such as into the bones, lungs or liver, survival is 11 percent (Cancer Centre 2022). Patients with TNBC experience distant recurrence more frequently (approximately 34% vs approximately 20%) and earlier (mean time to distant recurrence 2.6 years vs 5.0 years) compared with patients with other types of breast cancer (Dent R, 2007).	Disease pathology, burden of disease and recurrence risk.
		Metastatic triple negative breast cancer	TNBC is defined by a lack of progesterone receptors (PgR) and oestrogen receptors (ER), as well as the absence of human epidermal growth factor receptor 2 (HER2) overexpression or amplification (Dent R, 2007). TNBC accounts for approximately 10–15% of all breast cancers (Howard FM and Olopade OI, 2021; Bauer KR, 2007). Patients with TNBC experience distant recurrence more frequently (approximately 34% vs approximately 20%) and earlier (mean time to distant recurrence 2.6 years vs 5.0 years) compared with patients with other types of breast cancer (Dent R, 2007).	
**Cluster label: consumer comments**				
1293	Olaparib	Ovarian, fallopian tube or primary peritoneal cancer	The Medical Oncology Group of Australia (MOGA) also expressed its strong support for the olaparib submission, categorising it as one of the therapies of “highest priority for PBS listing” on the basis of the SOLO1 trial. The PBAC noted that the MOGA presented a European Society for Medical Oncology Magnitude of Clinical Benefit Scale (ESMO-MCBS) for olaparib, which was limited to 4 (out of a maximum of 5, where 5 and 4 represent the grades with substantial improvement), based on a comparison with placebo. The PBAC did not agree that this score should be updated from 3 to 4, as the PFS difference at 2 years was > 10%, but was not convincingly associated with a plateau of the PFS curve in the olaparib arm.	High need for listing, substantial clinical benefit.
		Ovarian cancer	The Medical Oncology Group of Australia (MOGA) expressed its strong support for the olaparib submission, categorising it as one of the therapies of “highest priority for PBS listing” on the basis of the PAOLA-1 trial. The PBAC noted that the MOGA presented a European Society for Medical Oncology Magnitude of Clinical Benefit Scale (ESMO-MCBS) for olaparib plus bevacizumab, of 4 (out of a maximum of 5, where 5 and 4 represent the grades with substantial improvement), based on a comparison with bevacizumab alone. The PBAC noted that MOGA upgraded the ESMO-MCBS score to 4 on the basis of a long term PFS difference >10% at 2 years and a plateau of the PFS curve in the treatment arm.	

Abbreviations: ADA; adalimumab, CMA; cost-minimisation approach, CZP; certolizumab pegol, ETN; etanercept, GOL; golimumab, GUS; guselkumab, IFX; infliximab, IXE; ixekizumab, PBAC; Pharmaceutical Benefits Advisory Committee, PFS; progression-free survival, SEC; secukinumab, TNBC; triple negative breast cancer, TOF; tofacitinib, UPA; upadacitinib, UST; ustekinumab.

Overall, the Euclidean distance analysis demonstrated different indications of multi-indication medicines were more semantically similar to each other than single-indication medicines. However, the cluster analysis showed that semantic overlap across all or similar indications of a medicine was infrequent.

## Discussion

The aim of the study was to identify the extent of semantic overlap between PSDs for different indications of the same medicine, namely as a means of identifying if semantic duplication exists across these PSDs and where this occurs so that this duplication could be eliminated as a way of streamlining the assessment of multi-indication medicines. While we observed higher semantic similarity within multi-indication medicines than across single-indication medicines, recurrence of the same concept across indications of a multi-indication medicine was infrequent. Therefore, it is unlikely that any concepts or information from assessments of medicines for previous clinical indications could be carried forward, whether manually or automatically, to the subsequent assessments of the medicine’s comparative safety, effectiveness and cost-effectiveness for other clinical indications.

### Interpretation of results

#### Euclidean distance analysis.

Across all PSD sections, we found greater semantic similarity across indications of the same medicine than across different medicines. The magnitude of difference across the two groups did vary by section (Fig 2).

Contrary to what we hypothesised, safety had the smallest difference between the two groups. This was disappointing, as we initially considered the assessment of medicine safety to be a promising component for carrying forward from previous indications to future assessments. During market authorisation, in some circumstances where medicines are being investigated for subsequent indications, the United States Food and Drug Administration allows for the collection of the safety data of a medicine to be less comprehensive or skipped altogether depending on the medicine’s existing safety profile [15]. We had anticipated that HTA might be able to adopt a similar approach. However, manual review of this section revealed that appropriately *different comparators* across indications resulted in distinct comparative safety evidence, limiting the feasibility of carrying forward the safety part of the assessment. This highlights key differences between regulatory bodies, focusing on *absolute* efficacy and safety, and HTA, which requires *comparative* evidence and an assessment of the magnitude of harms and benefits from the medicine.

The largest group difference occurred in the ‘population and disease’ section of the PSD. We did not anticipate this. As populations and diseases differ across indications of the same medicine, we expected similarity levels for this section to be comparable to the single-indication baseline. This result may primarily be driven by two factors. Firstly, this section does include a description of the medicine and its mechanism of action, and this will be essentially replicated across different indications of the same medicine. Second, different indications of the same medicine often belong to the same broad disease area, leading to overlaps in descriptions of disease pathology, burden, and clinical treatment pathways (this was evident when grouping indications based on AR-DRG codes).

We suspect that this second factor is also the reason why the clinical evidence section had the highest absolute similarity across all sections in the multi-indication medicine group (see Fig 2). In turn, this high similarity may be driven by discussion of the same clinical outcomes and terminology (e.g., progression-free survival and censoring for cancer medicines).

While these sections had high degrees of semantic similarity (in terms of Euclidean distance), using DBSCAN, we determined that this did not translate into semantically overlapping concepts across all or similar indications of multi-indication medicines. This suggests that, although text across indications often has similar terminology, the underlying concepts are often not duplicated (see Table 1). Consequently, low Euclidean distance scores at the section level reflect shared language patterns rather than a true recurrence of assessment content that could reduce duplication of effort and be carried forward.

#### Cluster analysis.

The cluster analysis using DBSCAN revealed a limited recurrence of concepts across all and similar indications of a medicine (Table 2). The most frequently overlapping clusters were labels corresponding to standard PSD template text. This was expected and does not represent meaningful duplication of assessment content. The second most frequent area of overlap was consumer comments, where the same concepts re-appeared in all indications in 11 of 59 multi-indication medicines. However, this means that, in 48 medicines (81%), consumer comments did not semantically overlap, ruling out the possibility of streamlining this section.

The lack of recurrence across the majority of multi-indication medicines in our dataset demonstrates that carrying forward (streamlining) is not possible, as semantically distinct concepts would likely be missed. For the purposes of our enquiry, we did not attempt to specify a set threshold of what level of recurrence would be sufficient to justify carrying forward assessment concepts. Our analysis demonstrates that no concepts recurred in more than 50% of multi-indication medicines, underscoring that there is insufficient evidence to justify removing or carrying forward any assessment concepts between indications.

Unsurprisingly, concept recurrence increased when examining similar indications within a medicine (F2), namely when the filtering requirement was relaxed from the concept needing to appear in *all* indications of a medicine to just *similar* indications of a medicine. However, the frequency of recurrence still remained well below the minimum level that could plausibly support any carrying forward of assessment components (i.e., 50%).

The relatively high recurrence of consumer comments was an important observation. Examination of the semantically overlapping texts revealed that this was largely driven by repeated references to “unmet clinical need”, specific clinical advantages of the medicine, and the use of specific rating scales, such as the European Society for Medical Oncology Magnitude of Clinical Benefit Scale. Because HTA is a relatively mature policy field, semantic overlap is easily detected where standardised terminology and assessment jargon are used across submissions [16].

This was reinforced during manual review of the DBSCAN clusters, as many clusters that were excluded or separated contained text that shared common terminology and discussed similar themes, but lacked true semantic overlap. While increasing the sensitivity of DBSCAN would have led to the automated exclusion of such clusters, this would also have increased the risk of excluding genuinely relevant overlap. The inflation of a similarity score between texts that share similar language but are semantically dissimilar (e.g., “*The PBAC recommended the listing*” vs. “*The PBAC did not recommend the listing*”) is a known limitation of SBERT embeddings [17]. However, because such cases were identified and excluded during manual review, this highlights the resilience of our combined NLP and manual review approach to address merely superficial similarity. Importantly, this known limitation does not affect the Euclidean distance analysis, as we deliberately focused on the difference in similarity from baseline (single-indication medicines), ensuring that any inflation in similarity was accounted for equally across groups. That is, the noise of any superficial similarity (similarity in text but not meaning) would have been on par across both groups, so subtracting one group from another would cancel out this noise.

### Implications for HTA practice

This study demonstrates that streamlining HTA for subsequent indications by carrying forward assessment content from previous indications is unlikely to be feasible. Our findings indicate that assessment content for a medicine is largely indication-specific, underscoring the continuing need for indication-based assessments. This position is consistent with guidance from the United States Food and Drug Administration on the use of Bayesian methods in clinical trials, which emphasises that borrowing evidence across related indications requires explicit justification of similarity and careful evaluation of prior-data conflict [18]. Even in regulatory settings which permit cross-indication borrowing, such approaches are conditional rather than automatic or routine. In the reimbursement context, this aligns with a previous analysis of PD-(L)1 checkpoint inhibitors submitted to the PBAC which identified substantial heterogeneity in clinical outcomes across different indications of the same medicine [19].

While this study focused on semantic overlap within HTA documents, future work could evaluate similarity across other components of HTA, such as clinical evidence packages or economic models in submissions. However, this would require access to confidential submission materials and the application of different methodological approaches capable of comparing clinical evidence or economic models. Identification of substantial similarity within these components may represent another avenue for streamlining other aspects of HTA processes for multi-indication medicines.

This study was conducted within the Australian PBAC context. As HTA typically “globalises the evidence and localises the decision”, assessment processes, evidentiary expectations and reporting practices may differ across jurisdictions [20]. Consequently, the extent of semantic overlap and the feasibility of streamlining may differ internationally. Nevertheless, the NLP workflow designed and implemented in this study provides a transferable methodological framework that other agencies could easily apply to their own assessments to evaluate the potential for streamlining.

Accelerating patient access is a recognised policy objective for HTA bodies globally [4,21]. As explained earlier, multi-indication medicines pose challenges in this regard, as each indication requires an assessment. Our findings, within the Australian setting, support that this assessment burden remains justified, given the limited recurrence of assessment content across indications. Consequently, if streamlining through carrying forward assessment content is not feasible, efficiency gains and faster patient access must be realised through alternative policy approaches. One such approach may be to optimise pricing and reimbursement arrangements across indications, especially considering that, internationally, patient access is predominantly delayed due to post-assessment activities, such as price negotiations [4]. Given that price-volume arrangements are considered simpler to implement for multi-indication medicines than pricing each indication separately, this may be a promising strategy for improving timely patient access [1].

In Australia, after several years of negotiations, the PBAC has accepted a broad multi-indication listing without the need for future HTAs on subsequent indications for two PD-(L)1 inhibitors, nivolumab and ipilimumab, under a price-volume arrangement [22]. Further work, including consultation with the wider HTA community, is needed to determine whether this approach can be applied more broadly to other multi-indication medicines.

### Methodological contributions and strengths

Building on our previous work in regulatory science, we demonstrate an NLP approach applied to HTA documents to identify and quantify degrees of semantic similarity and overlap [8–10]. To our knowledge, no other study has utilised SBERT and DBSCAN clustering on HTA documents.

Our findings did not support streamlining HTAs across indications of a medicine. Nevertheless, the methodological framework developed in this study can be applied to other areas where identification of textual and semantic similarity may either need to be identified or empirically ruled out. For example, the same approach could be applied to regulatory versus HTA documents to identify semantic overlap across the two, usually distinct processes, which may facilitate streamlining across these two pathways. Additionally, a retrospective review of HTA reports from different jurisdictions may uncover substantial semantic overlap, which would provide strong support for cross-jurisdictional assessment - a current priority of European HTA authorities [23]. Conversely, a finding of little semantic overlap (e.g., attributable to unique jurisdictional concerns) could indicate the need for a cautious approach to cross-jurisdictional assessment.

Beyond HTA, this methodology could be transferable to other policy areas where semantic overlap is suspected. For example, in environmental impact assessments, where the identification of potential duplication in assessments across agencies (such as state/provincial and federal governments) may provide further support for bilateral assessment approaches to minimize duplication and increase efficiency [24]. This approach could also have broader implications for benchmarking large language models by quantifying the semantic similarity between AI-generated and human-authored texts [25]. Human-authored texts may serve as the gold standard and similarity scores may serve as a quantitative measure of AI output quality (e.g., in answer to a question such as “What is HTA?”). However, as discussed earlier, the degree of standardisation in terminology is likely to influence these analyses. In fields where terminology is highly variable or inconsistently used, semantic overlap may be more difficult to detect because genuinely related concepts may be expressed in diverse ways and therefore appear more distant in the embedding space. Detecting such overlap may require less restrictive clustering parameters, which can increase the inclusion of unrelated text and require greater manual review. Conversely, fields with more established and consistently used terminology may be more amenable to this type of semantic similarity analysis.

Another methodological contribution from this research is the development of the post-hoc filters of DBSCAN clusters. While post-processing of DBSCAN clusters is standard practice, we applied dynamic deterministic filters specific to our research question. This allowed us to apply DBSCAN to the full dataset and thereby maximise the representation of the semantic space, i.e., provide the fullest possible picture of the different ways concepts were expressed across HTA documents. It also ensures internal consistency, as the outcome of different clustering runs cannot be easily compared. In effect, this approach improves clustering and allows us to focus manual efforts on data that were able to answer our research question [13].

A major strength of this study was that the NLP approach enabled a large-scale textual analysis, consistent with the broader trend towards using machine learning and advanced data analysis methods to examine complex datasets that are difficult or infeasible to review manually [26]. The use of the SBERT model means that this study is transparent and reproducible, which is why this approach was chosen over popular generative large language models (LLMs) such as ChatGPT and DeepSeek [27]. Additionally, generative processing of text does not easily translate to a quantitative analysis with statistically hard endpoints which may be needed to inform policy development. Nevertheless, LLMs could complement the current framework by assisting with qualitative interpretation, for example proposing provisional labels or summarising the content of clusters. This may be particularly useful when the number of clusters exceeds the capacity for detailed manual review.

Our statistical analysis plan for comparing Euclidean distances was tailored to our specific research question and approach. A key uncertainty in this approach concerned the one versus two step averaging framework which we had addressed by conducting a bootstrap sensitivity analysis. Finally, for DBSCAN, we systematically fine-tuned ε, in increments of 0.01, with multiple evaluation indices and manual review to identify a value that balanced sensitivity and specificity in our dataset.

All of these novelties advance methods in the use of NLP to compare texts for semantic similarity, remembering that these methods are applicable well beyond HTA and could even play a key role in benchmarking LLMs for retrieval or comparison tasks relying on semantic similarity.

### Limitations

The main limitation of our study is the data source. As mentioned previously, the PSDs, whose role is to summarise appraisal decision making and not necessarily the full-length technology evaluation proper, are the only publicly available HTA document in Australia due to commercial-in-confidence requirements. It is possible that we would have uncovered greater semantic overlap in actual HTA reports or submission dossiers. It is also possible that some detailed aspects of assessment content, such as nuanced safety considerations or technical modelling critiques, may not be fully represented within the PSDs. Limitations also exist within the SBERT model, as it inherits biases from its training data, general purpose corpora which are not necessarily specific to HTA, regulatory or biomedical documents. Although there are other BERT models developed for these areas (PubMedBERT and PharmBERT), the literature suggests SBERT is still the best embedding model for similarity tasks [8,11]. Finally, our paragraph-based chunking approach may have also introduced some bias. While typically a distinct concept is discussed in each paragraph of a PSD, occasionally multiple concepts are discussed, which could have reduced the precision of SBERT embeddings by blending distinct concepts into one vector representation [17]. However, this would have minimal impact on the Euclidean distance analysis, as that analysis is based on the average pairwise distances across many text chunks, where small embedding inaccuracies are diluted. By contrast, this limitation may have a greater effect on DBSCAN clustering, which is more sensitive to small variations between individual embeddings.

## Conclusions

We aimed to examine whether streamlining of the HTA assessment process for multi-indication medicines could be achieved by identifying duplicative areas and concepts that could be carried forward from previous indications to subsequent indications of a medicine when they are assessed. We designed and implemented an NLP approach to quantify the degrees of semantic similarity and overlap between HTA documents of different indications of a medicine versus HTA documents of different medicines. Higher semantic similarity was observed within multi-indication medicines than across different medicines. However, the absolute amount of semantic overlap within multi-indication medicines was too low to be of practical utility for streamlining. The semantic similarity observed was driven by shared language patterns rather than true recurrence of assessment content of the sort which could be carried forward across indications of the same medicine. Based on these findings, we consider that streamlining of HTAs across multiple indications for a medicine is not viable. The methods described in this study provide a foundation for future research on semantic overlap across other documents in HTA and across other policy areas where semantic overlap may be helpful to identify or rule out.

## Supporting information

S1 File Supplementary methods and results. This file provides additional technical detail to complement the methods and results sections of the main manuscript.
(DOCX)

S2 File Cluster members, AR-DRG groupings, Euclidean distances. This file provides the cluster members (i.e., text chunks) that were used during the cluster analysis and thematic review (sheet 1), the AR-DRG codes used for F2 clustering and the full data set for Euclidean distances depicted in Fig 2.
(XLSX)
