# Supplementary material for: A natural language processing approach to determine whether streamlined health technology assessment for multi-indication medicines is feasible
Source: PLOS Digit Health. 2026 Aug 26;5(8):e0001657. doi: 10.1371/journal.pdig.0001657 (PMC13514061; doi:10.1371/journal.pdig.0001657)
Supplement: Supplementary file 1 [file pdig.0001657.s001.docx]

## Additional File 1 for

# A Natural Language Processing Approach to Determine Whether Streamlined Health Technology Assessment for Multi-Indication Medicines is Feasible

**Authors:**

Isaiah Luc^1^

Drew Carter^1^

Erik Bergman^2^

Gabriel Westman^2,3^

Tracy Merlin^1^

^1^ Adelaide Health Technology Assessment, School of Public Health, Adelaide University, Adelaide, South Australia, Australia

^2^ Swedish Medical Products Agency, Uppsala, Sweden

^3^ Department of Medical Sciences, Uppsala University, Uppsala, Sweden

**Corresponding Author:**

Isaiah Luc

Email: Isaiah.Luc@adelaide.edu.au

### Supplementary Methods: Additional Technical Detail

These methods provide additional technical detail to complement the Methods section in the main manuscript.

#### Data acquisition and pre-processing

The Pharmaceutical Benefits Scheme (PBS) Medicine Status Lookup website lists all medicine submissions considered by the Pharmaceutical Benefits Advisory Committee (PBAC) since July 2019, including the type of submission, the indication and the meeting date.^1^ This resource was used to screen each submission against our inclusion and exclusion criteria.

The corresponding Public Summary Document (PSD) for each included submission was then manually downloaded (as .docx files) from the PBS website and each PSD was checked to ensure it met the inclusion criteria (as sometimes the actual PSD differed to what was reported on the PBS Medicine Status website).

PSDs were prepared by removing tables, figures and table and figure captions and notes, because we wanted to limit the analysis to the body text of the PSD. All downloaded PSDs were converted into plain-text format for processing. Text files were automatically sectioned into: population and disease, comparator, clinical evidence, safety, economic evaluation, financial implications, PBAC outcome and the full document based on template headings in the PSDs (see Table S1).

Each section was further segmented into chunks at the paragraph level. However, paragraphs consisting of a single sentence or less were excluded as typically these are part of standard templated text. Paragraphs exceeding 200 words were further divided into chunks of up to 200 words, with a 50-word overlap between consecutive segments. The 200-word limit was chosen to remain within the maximum input length of the Sentence-Bidirectional Encoder Representations from Transformers (SBERT) model and hence avoid truncation of text during embedding. This approach was adapted from the previously used method in our full-document semantic matching analysis.^2^

#### Euclidean distance analysis

To summarise the similarity between two documents, we applied a mean of minimum approach. That is, for each chunk in one section, the most similar chunk in the same section of the other PSD was identified (based on the minimum Euclidean distance), and the average of these minimum values was computed. As the results are not symmetrical (i.e., PSD A vs PSD B yielded different results from PSD B vs PSD A), this procedure was performed in both directions, and the two means were averaged. This method captured the strongest semantic overlap between documents while minimising the influence of non-matching or unrelated content and was previously used in our full document semantic matching analysis.^2^

#### Cluster analysis

Embeddings from the safety and full document section were excluded from the Density-Based Spatial Clustering of Applications with Noise (DBSCAN) as these embeddings are already included in other sections.

In terms of parameter selection for epsilon (ε), candidate values of ε were guided by inspection of the k-distance curve (k=2, aligned with the minimum samples value) and evaluation of standard clustering validity indices (Silhouette coefficient, Davies–Bouldin index, and Calinski–Harabasz score).^3^ In addition, two-dimensional t-Distributed Stochastic Neighbour Embedding (t-SNE) projections were generated to visualise the embedding space under different parameterisations. Finally manual inspection of each cluster was conducted to confirm semantic consistency.

#### Sensitivity analyses

For the bootstrap sensitivity analysis, random pseudo-groups of single-indication PSDs with group sizes matched to those of the multi-indication medicines were generated. For each iteration, pseudo-groups were sampled without replacement within medicines and with replacement across medicines as needed. The mean pairwise Euclidean distance was calculated within each pseudo-group, and these group means were averaged to yield a single statistic for that iteration. This process was repeated 10,000 times per section to produce a bootstrap null distribution. Empirical p-values and 95% percentile intervals were derived by comparing the observed single-indication mean to this null distribution, to assess whether the averaging framework used for multi-indication medicines introduced bias.

### Supplementary Results: Additional Technical Detail

*Ε parameter selection*

The k-distance curve displayed its first bend just before 0.4, with the slope increasing steadily until just before 0.5 (see Figure S1). This elbow pattern suggested that ε values in the range of 0.4–0.5 were optimal for distinguishing semantically aligned clusters from noise. Based on this observation, we systematically tested ε values within this range in increments of 0.01. Clustering metrics suggested that a lower ε produced the most distinct clusters, however this also resulted in a very high proportion of noise (>85%), leaving few usable clusters (Table S4). Manual review of the meaning of different text clusters revealed that at ε = 0.45, clusters contained a broad range of semantically unrelated content. By contrast, ε = 0.44 produced clusters that were more semantically aligned, accordingly, 0.44 was selected as the most appropriate value for cluster labelling.

### Supplementary Tables

**Table S1: Definitions of section boundaries used to section PSDs**

| **Section Name** | **Section starter** | **End of section** |
| --- | --- | --- |
| Population and Disease | "Population and disease" | "Comparator" |
| Comparator | "Comparator" | "Consideration of the evidence" |
| Clinical Evidence of Effectiveness | "Consideration of the evidence" | "Economic analysis" |
| Safety | “Comparative harms” | “Clinical claim” |
| Economic Analysis | "Economic analysis" | "Estimated PBS usage & financial implications" |
| Financials Implications | "Estimated PBS usage & financial implications" | "PBAC outcome" |
| PBAC Outcome | "PBAC outcome" | "Context for Decision" |
| Full Document | Entire document | |

Abbreviations: PBAC; Pharmaceutical Benefits Advisory Committee, PBS; Pharmaceutical Benefits Scheme, PSD; Public Summary Document.

Notes: Sections were extracted from each PSD by identifying the first occurrence of the specified start and end headings and retrieving all narrative text appearing between them. The “Full Document” section included the entire document.

**Table S2: Independent sample t-tests comparing Euclidean distances between single and multi-indication medicines across PSD sections**

| **Section** | **Group** | **n** | **Mean** | **CI** | **Mean difference** | **95% CI** | **T statistic** | **P value** |
| --- | --- | --- | --- | --- | --- | --- | --- | --- |
| Population and Disease | Single | 11,628 | 1.181 | 1.18, 1.183 | 0.338 | 0.301, 0.375 | 18.356 | 1.19E-25 |
|  | Multi | 58 | 0.843 | 0.806, 0.880 |  |  |  |  |
| Comparator | Single | 10,878 | 1.043 | 1.041, 1.044 | 0.192 | 0.162, 0.221 | 12.899 | 1.35E-18 |
|  | Multi | 58 | 0.851 | 0.822, 0.881 |  |  |  |  |
| Clinical Evidence of Effectiveness | Single | 11,781 | 0.991 | 0.990, 0.992 | 0.204 | 0.188, 0.220 | 25.109 | 5.33E-33 |
|  | Multi | 59 | 0.787 | 0.771, 0.803 |  |  |  |  |
| Safety | Single | 11,325 | 1.001 | 1.000, 1.002 | 0.162 | 0.136, 0.188 | 12.419 | 5.17E-18 |
|  | Multi | 59 | 0.839 | 0.813, 0.865 |  |  |  |  |
| Economic Analysis | Single | 11,628 | 0.981 | 0.98, 0.982 | 0.169 | 0.146, 0.192 | 14.662 | 3.24E-21 |
|  | Multi | 59 | 0.811 | 0.788, 0.835 |  |  |  |  |
| Financial Implications | Single | 11,781 | 0.994 | 0.993, 0.995 | 0.164 | 0.142, 0.186 | 14.658 | 3.40E-21 |
|  | Multi | 59 | 0.830 | 0.808, 0.853 |  |  |  |  |
| PBAC outcome | Single | 11,781 | 0.984 | 0.982, 0.985 | 0.226 | 0.203, 0.248 | 19.761 | 1.56E-27 |
|  | Multi | 59 | 0.758 | 0.735, 0.781 |  |  |  |  |
| Full Document | Single | 11,781 | 0.951 | 0.95, 0.952 | 0.212 | 0.197, 0.228 | 27.638 | 3.39E-35 |
|  | Multi | 59 | 0.738 | 0.723, 0.754 |  |  |  |  |

Abbreviations: CI; confidence interval, PBAC; Pharmaceutical Benefits Advisory Committee, PSD; Public Summary Document.

**Table S3: Bootstrap sensitivity analysis of semantic similarity by PSD section**

| **Section** | **Observed mean**  **(single-indication medicines)** | **Null mean** | **Null SD** | **Null 95% CI** | **P value** |
| --- | --- | --- | --- | --- | --- |
| Population and Disease | 1.181 | 1.181 | 0.008 | 1.165, 1.195 | 0.995 |
| Comparator | 1.043 | 1.043 | 0.007 | 1.028, 1.056 | 0.999 |
| Clinical Evidence of Effectiveness | 0.991 | 0.991 | 0.004 | 0.983, 0.999 | 0.980 |
| Safety | 1.001 | 1.001 | 0.005 | 0.991, 1.011 | 0.993 |
| Economic Analysis | 0.981 | 0.981 | 0.005 | 0.971, 0.991 | 1.000 |
| Financial Implications | 0.994 | 0.994 | 0.004 | 0.986, 1.002 | 0.990 |
| PBAC outcome | 0.984 | 0.984 | 0.006 | 0.972, 0.995 | 0.997 |
| Full Document | 0.951 | 0.951 | 0.004 | 0.944, 0.958 | 0.989 |

Abbreviations: CI; confidence interval, PBAC; Pharmaceutical Benefits Advisory Committee, PSD; Public Summary Document, SD; standard deviation.

Notes: Observed mean is the mean Euclidean distance for the observed data. The null mean, null SD and null 95% CI describe the bootstrap distribution generated from pseudo-groups of single-indication PSDs.

**Table S4: DBSCAN clustering characteristics and evaluation metrics across candidate ε values**

| **ε** | **Number of clusters** | **% noise^a^** | **Silhouette coefficient** | **Davies-Bouldin index** | **Calinski-Harabasz score** |
| --- | --- | --- | --- | --- | --- |
| 0.40 | 1,258 | 87% | 0.563 | 0.589 | 34.090 |
| 0.41 | 1,384 | 86% | 0.538 | 0.624 | 30.317 |
| 0.42 | 1,485 | 85% | 0.515 | 0.658 | 27.558 |
| 0.43 | 1,630 | 83% | 0.490 | 0.691 | 25.023 |
| 0.44 | 1,718 | 82% | 0.465 | 0.735 | 22.576 |
| 0.45 | 1,853 | 80% | 0.439 | 0.769 | 20.749 |
| 0.46 | 1,978 | 78% | 0.415 | 0.811 | 19.139 |
| 0.47 | 2,093 | 76% | 0.392 | 0.868 | 17.811 |
| 0.48 | 2,170 | 74% | 0.366 | 0.911 | 16.574 |
| 0.49 | 2,281 | 72% | 0.343 | 0.961 | 15.482 |
| 0.50 | 2,358 | 69% | 0.315 | 1.030 | 14.189 |

Abbreviations: ε; epsilon.

Note: Minimum samples was set to 2.

^a^ Refers to the percentage of text chunks which were not assigned to any cluster.

### Supplementary Figures

**Figure S1: K-distance plot (k = 2) used to guide selection of DBSCAN parameter ε.**

Abbreviations: ε; epsilon, DBSCAN; Density-Based Spatial Clustering of Applications with Noise.

Notes: The elbow region (0.4 - 0.5) informed the testing of candidate ε values.

**Figure S2: t-SNE projection of all text embeddings with DBSCAN cluster labels, noise included**

Abbreviations: DBSCAN; Density-Based Spatial Clustering of Applications with Noise, t-SNE; t-Distributed Stochastic Neighbour Embedding, SBERT; Sentence-Bidirectional Encoder Representations from Transformers.

Notes: Points represent paragraph-level text chunks embedded with SBERT. Colours indicate DBSCAN clusters, grey points represent noise. The plot illustrates the full structure of the embedding space prior to any filtering.

**Figure S3: t-SNE projection of all text embeddings with DBSCAN cluster labels, noise removed**

Abbreviations: DBSCAN; Density-Based Spatial Clustering of Applications with Noise, t-SNE; t-Distributed Stochastic Neighbour Embedding, SBERT; Sentence-Bidirectional Encoder Representations from Transformers.

Notes: Points represent paragraph-level text chunks embedded with SBERT. Colours indicate DBSCAN clusters.

**Figure S4: Cluster inclusion and exclusion workflow after DBSCAN**

Abbreviations: DBSCAN, Density-Based Spatial Clustering of Applications with Noise.

Note: cluster members refer to only those members who contributed to the filtering predicates. F1 (full-medicine coverage): keep clusters that include ≥1 text chunk from every indication of a multi-indication medicine. F2 (similar-indication coverage): within each medicine, keep clusters that include ≥1 text chunk from every indication in a group of similar indications (ignore groups of size 1).

### References

1.	Australian Government Department of Health, Disability and Ageing. Medicine Status Lookup. Accessed 30 June, 2025. <https://www.pbs.gov.au/medicinestatus/search.html?sort=psproperty-drug-name>

2.	Bergman E, Pasmooij AMG, Mol PGM, Westman G. A full-document analysis of the semantic relation between European Public Assessment Reports and EMA guidelines using a BERT language model. *PLOS ONE*. 2023;18(12):e0294560. doi:10.1371/journal.pone.0294560

3.	Schubert E, Sander J, Ester M, Kriegel HP, Xu X. DBSCAN Revisited, Revisited: Why and How You Should (Still) Use DBSCAN. *ACM Trans Database Syst*. 2017;42(3):Article 19. doi:10.1145/3068335
