# Supplementary material for: A natural language processing approach to determine whether streamlined health technology assessment for multi-indication medicines is feasible
Source: PLOS Digit Health. 2026 Aug 26;5(8):e0001657. doi: 10.1371/journal.pdig.0001657 (PMC13514061; doi:10.1371/journal.pdig.0001657)
Supplement: Supplementary file 2 [file pdig.0001657.s004.docx]

**Response to Reviewers**

**Manuscript Number: PDIG-D-26-00248**

**Manuscript Title: A Natural Language Processing Approach to Determine Whether Streamlined Health Technology Assessment for Multi-Indication Medicines is Feasible**

Dear Dr Luo, Dr Yoon and Dr Celi

We would like to thank you and the reviewers for the time and effort dedicated to providing constructive feedback on our manuscript. We have carefully considered all comments and made revisions to the manuscript in response.

We believe the revisions made have significantly strengthened the manuscript. A detailed, point-by-point response is attached below, outlining how each reviewer comment has been addressed.

We sincerely appreciate the reviewers’ insights and your consideration of our resubmission. We hope that the revised manuscript now meets the standards for publication in *PLOS Digital Health.*

Yours sincerely,

Mr Isaiah Luc

Dr Drew Carter

Mr Erik Bergman

A/Prof Gabriel Westman and

Prof Tracy Merlin

**Reviewer 1:**

| ***Reviewer comment*** | **Authors’ comment** | **Changes in manuscript** |
| --- | --- | --- |
| *This study relies on Public Summary Documents (PSDs) instead of complete assessment reports, which is a significant limitation. While the authors note that PSDs are "the best available option," these summaries may omit important details from the full reports.* | We agree this is an important limitation to highlight in the manuscript (which we have highlighted 148 - 150 and 515 -519).  However, PSDs reflect key decision-making considerations underpinning PBAC recommendations. Therefore they represent an important starting point for examining whether assessment content is consistent across indications and whether any content could potentially be reused.  Additionally, we would like to reassure that, while PSDs are summary documents, they are often extensive and detailed documents (in some cases exceeding 70 pages), rather than brief condensed summaries that omit substantial aspects of the assessment process. | We have added additional justification for using PSDs in the methods section of the manuscript to reassure the reader that PSDs are extensive detailed documents (148 – 154)  We have also added an additional sentence in the limitations section that further highlights that detailed assessment content may not be fully represented within the PSDs (see 502 – 504). |
| *The study focuses mainly on repeated text. However, streamlining also includes reusing clinical or economic models, which may not be captured by analyzing paragraphs alone.* | We would like to clarify that this study did not focus solely on repeated text, but rather on semantic similarity and semantic overlap, namely whether written assessment content conveyed similar underlying *meaning* across indications.  However, we do agree that streamlining could also occur by carrying forward clinical or economic models, not just meaning embedded in text. However, model details are not publicly available. Furthermore, this falls outside the design of our study. A completely different study design, in addition to new, non-public data sources, would be needed to assess the potential for model carry-forward. Thus, we must simply acknowledge this as a limitation of the study.  Our methods represent a necessary and useful first step in assessing potential opportunities for streamlining using publicly available data. | To acknowledge the reviewer’s broader perspective, we have added text to the Discussion section noting that future work could evaluate semantic similarity and overlap across these components to determine whether they may represent additional opportunities for streamlining aspects of HTA processes for multi-indication medicines, noting that different methods are needed (see 428 - 432). |
| *The analysis is limited to the Australian Pharmaceutical Benefits Advisory Committee (PBAC) context. While this provides a solid foundation, the findings may not apply to other HTA regions with different reporting practices.* | We agree that this study, conducted in the Australian context, provides a solid foundation.  While these findings may not be directly transferable to all HTA jurisdictions, the methodological framework developed in this study remains relevant for HTA systems who may wish to consider semantic similarity among their own policy documents. | In the discussion section we have clarified that implications may differ across jurisdictions, however we note that the NLP workflow can be easily implemented in other jurisdictions so they can evaluate their own potential for streamlining (see 438 – 444). |
| *In the Introduction and in the Methodology. It is suggested to refer from the paper: “Systematic Analysis Based on the Confluence of Machine Learning and the Internet of Things Using Bibliometric Analysis. It is important to support the growing role of advanced data analysis and machine learning in examining large datasets. This reinforces the paper’s argument by placing the use of Natural Language Processing (NLP) within the broader trend of using technology to analyze complex data that is difficult to study manually.* | We agree that it is useful to situate our use of NLP within the broader trend of applying machine learning and advanced data analysis methods to complex datasets that are difficult to examine manually. | We have therefore added a brief comment relating to this in our discussion (see 496 – 497). |
| *The authors should explain why PSDs are enough for finding streamlining opportunities. They need to consider whether summarization might omit critical details, such as safety data, that are important to retain.* | As raised above, we acknowledge the reviewer’s concerns. PSDs reflects the key decision-making rationale for HTA applications (see 148 – 150). | As mentioned above, we have added a sentence in the limitations section that discusses that detailed assessment content may not be fully represented within the PSDs (see 519 - 521). |
| *The conclusion that streamlining is "unlikely" based on infrequent recurrence needs a more thorough discussion. Just because certain concepts occur infrequently does not mean specific sections, like safety, cannot be reused. The authors should check if some sections show higher recurrence than others.* | We thank the reviewer for this comment and the opportunity clarify. We believe there is some misunderstanding on the part of the reviewer.  We have interpreted this comment as raising the concern that, even where recurrence is infrequent overall, specific sections such as safety may still contain reusable content. However, our conclusion was based on recurrence of semantically overlapping concepts, which are more granular than whole sections. A section could only plausibly be reused if it contained recurring concepts or assessment content that could be carried forward. Therefore, the low recurrence of individual semantically overlapping concepts within our dataset, including safety-related concepts, does support our conclusion that content cannot be routinely carried forward without risk.  Our cluster analysis was conducted to identify specific text chunks where concepts (i.e., meaning) recurred across indications of the same medicine. This is because such recurrence could plausibly represent assessment content suitable for carrying forward. Importantly, recurrence was evaluated across entire documents rather than separately within predefined sections, meaning that any genuinely recurring concepts from sections, such as safety, were still able to be identified through the clustering process. While some sections demonstrated greater semantic similarity at the section level, the number of recurring (semantically overlapping) concepts identified through clustering remained low (Table 2). In short, there just wasn’t much repetition of ideas. We looked very carefully for it.  This is explained in detail in the manuscript (see 359 – 363). | We have added text to further clarify the intention of the cluster analysis in the Methods section (see 204 – 207). |

**Reviewer 2**

| ***Reviewer comment*** | **Authors’ comment** | **Changes in manuscript** |
| --- | --- | --- |
| *Using natural language processing (NLP) in finding patterns and concepts in medical and health technology texts is a timely application of this technology and there is a great potential that NLP would help in streamlining the repetitive manual processes. This manuscript could be bases for future research in this subject as concluded in the abstract.* | Thank you, we agree. | No changes necessary. |
| *The abstract is following the standards of giving the research background, methods, results and conclusion.* | Thank you, we agree. | No changes necessary. |
| *“Beyond this specific application, our approach demonstrates how machine learning can be used to measure similarity across other types of health policy documents.” I agree that this statement is very important. Even more important than the hypothesis behind the manuscript, which proved to be not valid, about the use of similarity of document contents across multiple indications for the same medicine.* | Thank you, we agree. | No changes necessary. |
| *“Such methods could help policymakers determine other processes that can be streamlined.” For wider audience of readers, could you please provide one example?* | We agree. We have added some examples to the Author Summary. A more detailed discussion is provided in the Discussion section of the manuscript. | We have revised the Author Summary to include examples of how the NLP framework could be applied beyond the current study context (see line 65). |
| *“Statistical analysis” This heading needs to bold or underscored.* | Agreed. We welcome further formatting advice from the editor. | We have underlined this heading, and other headings with the same level in the manuscript. |
| *“Cluster filtering and labelling” That is a thorough process to ensure that every source of noise is excluded.* | Thank you. | No changes necessary. |
| *The manuscript provided for review is not showing the figures, tables in the right inserts, which made the review a bit of a harder job.* | We apologies for the formatting error. We have ensured the formatting of the manuscript is correct and we welcome any further advice from the editor. | We have ensured proper formatting through the manuscript. |
| *“Consequently, for faster access to be achieved, efficiency gains must be realised through alternative approaches.” The approaches suggested from here down are not technological alternatives to NLP, rather they are practical approaches to solve the studied problem. As a reader, I would expect that the focus throughout the manuscript to be on finding a solution for the streamlining problem rather than to find a specific technological solution.* | We agree that the primary focus of the manuscript is the broader policy question of whether HTA for multi-indication medicines can be streamlined.  In our study, NLP was used as a methodological tool to investigate whether semantic overlap existed across HTA documents in a way that could support carrying forward assessment content between indications.  We believe broader discussion (i.e. the alternative policy approaches suggested) are important and relevant for HTA readers, as the ultimate objective is to identify practical approaches that may reduce system burden while maintaining robust assessment standards.  The methodological refinement of NLP approaches to policy and other areas is a secondary, still-very-important focus of the manuscript. | We have revised the Discussion section to clarify that the subsequent approaches discussed (e.g., pricing and reimbursement arrangements) are not presented as alternatives to NLP, but rather as alternative policy approaches that may improve efficiency and patient access when streamlining assessment through carrying forward assessment content appears infeasible (see 437 – 439).  We also added discussion noting that further investigation for streamlining could evaluate other components of HTA, such as clinical evidence packages or economic models, as per the reviewer’s suggestion (see 428 – 432). |
| *“the methodological framework developed here is able to be applied to other areas where identification of text and semantic similarity is needed.” If the methodological framework did not prove itself in the situation in hand, how is it expected to be taken forward for other area of application?* | We thank the reviewer for this comment. We would like to clarify that the methodological framework itself performed as intended and successfully addressed the research question by identifying and quantifying semantic similarity and overlap across HTA documents. Importantly, the finding that semantic overlap was insufficient to support carrying forward assessment content is itself a meaningful result enabled by the NLP framework, rather than a failure of that framework, as it provides empirical evidence challenging previous hypotheses that substantial similarity exists across indications of the same medicine (see Background).  This study was not intended to serve as a validation study of the NLP methods themselves, but rather builds upon previous published work which has already successfully applied SBERT and clustering approaches to identify semantic similarity and overlap within regulatory and policy documents (see 132 – 138 and 451). The study was primarily designed to apply NLP methods, not evaluate or prove them.  Additionally, our workflow incorporated a manual validation step through cluster filtering and labelling, whereby potentially semantically overlapping text chunks identified by DBSCAN were manually reviewed (eyeballed) to confirm genuine semantic overlap through human judgement (more detail is provided in the Additional Files). As the reviewer also acknowledged elsewhere, this combined NLP and manual review approach represents a thorough methodology for evaluating semantic similarity across policy documents.  An important contribution of this work is demonstrating how machine learning approaches can be used to measure similarity across health policy documents and potentially other documents. The Discussion section is intended to explain the potential implications and applicability of this framework to other policy and document analysis contexts. | We have revised the Discussion section to further clarify that the framework remains valuable irrespective of whether semantic overlap is ultimately identified or ruled out (see 450 - 453) |
| *“Additionally, a retrospective review of HTA reports from different jurisdictions may uncover substantial semantic overlap” Could you provide the reader with a justification for this assumption?* | We thank the reviewer for this comment. We would like to clarify that this statement was not intended to assume that substantial semantic overlap necessarily exists across HTA reports from different jurisdictions. The statement was intended as a conjecture or hypothesis for future investigation.  Importantly, the next sentence in the manuscript outlines both possible outcomes of such an analysis: either substantial semantic overlap may be identified, supporting opportunities for cross-jurisdictional assessment, or limited semantic overlap may be observed, suggesting that jurisdiction-specific considerations remain important (see 463 – 465). Our intention was therefore to highlight the applicability of the framework and potential implications, not make an assumption regarding expected findings. | No changes necessary. |
| *“The more mature the field is, the more useful our method may be, since agreed terminology may recur more often.” What are the manuscript’s criteria to define ‘field maturity’?* | We agree that the phrase “field maturity” was imprecise and may imply a formally defined construct, which was not intended in this context.  The intention of this statement was to outline this was an observation arising from our findings and manual review of clusters. | We have revised the wording in the manuscript to instead refer to the degree of standardisation in terminology and have clarified our position behind this statement (see 486 – 496). |
| *“NLP to compare texts for semantic similarity, remembering that these methods are applicable well beyond HTA and could even play a key role in benchmarking LLMs.” I am sure that you have reached this conclusion with elaborate efforts, but it needs to be more convincingly clear to the reader.* | We thank the reviewer for this comment. This sentence was intended to be a summary sentence based on the previous paragraphs, hence we believe this point is already thoroughly explained in the above discussion, where we outline how our methodological approach can assist with LLM benchmarking (see 462 - 466). | We have added some more detail to this sentence to further clarify our position (see 520 - 521) |
| *Thanks again. In this review, the focus is on the reader understanding and expanding the target audience of the manuscript. The questions are genuinely based on the reader trying to understand the text and connecting the dots in the information conveyed in it.*  *I appreciate the research efforts and all the work put into the study and I repeat my gratitude to be given this opportunity.* | We thank the reviewer for their thoughtful and constructive feedback. Our intention was to present the study in a way that allows readers from a broad range of backgrounds, including those less familiar with NLP methodologies, to follow the rationale, connect the findings, and understand the wider implications of the work. | No changes necessary. |
