# Supplementary material for: A natural language processing approach to determine whether streamlined health technology assessment for multi-indication medicines is feasible
Source: PLOS Digit Health. 2026 Aug 26;5(8):e0001657. doi: 10.1371/journal.pdig.0001657 (PMC13514061; doi:10.1371/journal.pdig.0001657)
Supplement: Peer review history

# Peer review: Decision Letter 0

**DOI:** 10.1371/journal.pdig.0001657.r001

**Authors:** Dukyong Yoon, Peng Luo

27 Apr 2026

Response to Reviewers Revised Manuscript with Track Changes Manuscript

**Journal Requirements:**

1. Please send a completed 'Competing Interests' statement, including any COIs declared by your co-authors. If you have no competing interests to declare, please state "The authors have declared that no competing interests exist". Otherwise please declare all competing interests beginning with the statement "I have read the journal's policy and the authors of this manuscript have the following competing interests:"

2. We note that your Data Availability Statement is currently as follows: The datasets supporting the conclusions of this article are included within the article and its additional files.

Please confirm at this time whether or not your submission contains all raw data required to replicate the results of your study. Authors must share the “minimal data set” for their submission. PLOS defines the minimal data set to consist of the data required to replicate all study findings reported in the article, as well as related metadata and methods (https://journals.plos.org/plosone/s/data-availability#loc-minimal-data-set-definition).

For example, authors should submit the following data:

- The values behind the means, standard deviations and other measures reported;

- The values used to build graphs;

- The points extracted from images for analysis.

Authors do not need to submit their entire data set if only a portion of the data was used in the reported study.

If your submission does not contain these data, please either upload them as Supporting Information files or deposit them to a stable, public repository and provide us with the relevant URLs, DOIs, or accession numbers. For a list of recommended repositories, please see https://journals.plos.org/plosone/s/recommended-repositories.

If there are ethical or legal restrictions on sharing a de-identified data set, please explain them in detail (e.g., data contain potentially sensitive information, data are owned by a third-party organization, etc.) and who has imposed them (e.g., an ethics committee). Please also provide contact information for a data access committee, ethics committee, or other institutional body to which data requests may be sent. If data are owned by a third party, please indicate how others may request data access.

3. Please upload separate figure files in .tif or .eps format. Also, remove the figures from your manuscript file but keep the legends.

For more information about figure files please see our guidelines:

https://journals.plos.org/digitalhealth/s/figures

https://journals.plos.org/digitalhealth/s/figures#loc-file-requirements

4. We have noticed that you have uploaded Supporting Information files, but you have not included a list of legends. Please add a full list of legends for your Supporting Information files after the references list.

**Additional Editor Comments (if provided):** **Reviewers' Comments:**

**Comments to the Author**

1. Does this manuscript meet PLOS Digital Health’s publication criteria?

Reviewer #1: Yes

Reviewer #2: Partly

**********

2. Has the statistical analysis been performed appropriately and rigorously?

Reviewer #1: Yes

Reviewer #2: Yes

**********

3. Have the authors made all data underlying the findings in their manuscript fully available (please refer to the Data Availability Statement at the start of the manuscript PDF file)?

The PLOS Data policy

Reviewer #1: Yes

Reviewer #2: Yes

**********

4. Is the manuscript presented in an intelligible fashion and written in standard English?

Reviewer #1: Yes

Reviewer #2: Yes

**********

**Reviewer #1:**  1. This study relies on Public Summary Documents (PSDs) instead of complete assessment reports, which is a significant limitation. While the authors note that PSDs are "the best available option," these summaries may omit important details from the full reports.

2. The study focuses mainly on repeated text. However, streamlining also includes reusing clinical or economic models, which may not be captured by analyzing paragraphs alone.

3. The analysis is limited to the Australian Pharmaceutical Benefits Advisory Committee (PBAC) context. While this provides a solid foundation, the findings may not apply to other HTA regions with different reporting practices.

4. In the Introduction and in the Methodology. It is suggested to refer from the paper: “Systematic Analysis Based on the Confluence of Machine Learning and the Internet of Things Using Bibliometric Analysis. . It is important to support the growing role of advanced data analysis and machine learning in examining large datasets. This reinforces the paper’s argument by placing the use of Natural Language Processing (NLP) within the broader trend of using technology to analyze complex data that is difficult to study manually.

5. The authors should explain why PSDs are enough for finding streamlining opportunities. They need to consider whether summarization might omit critical details, such as safety data, that are important to retain.

6. The conclusion that streamlining is "unlikely" based on infrequent recurrence needs a more thorough discussion. Just because certain concepts occur infrequently does not mean specific sections, like safety, cannot be reused. The authors should check if some sections show higher recurrence than others.

**Reviewer #2:**  Thanks for giving me the opportunity to review this manuscript. Please find my comments below:

• Using natural language processing (NLP) in finding patterns and concepts in medical and health technology texts is a timely application of this technology and there is a great potential that NLP would help in streamlining the repetitive manual processes. This manuscript could be bases for future research in this subject as concluded in the abstract.

• The abstract is following the standards of giving the research background, methods, results and conclusion.

• “Beyond this specific application, our approach demonstrates how machine learning can be used to measure similarity across other types of health policy documents.” I agree that this statement is very important. Even more important than the hypothesis behind the manuscript, which proved to be not valid, about the use of similarity of document contents across multiple indications for the same medicine.

• “Such methods could help policymakers determine other processes that can be streamlined.” For wider audience of readers, could you please provide one example?

• “Statistical analysis” This heading needs to bold or underscored.

• “Cluster filtering and labelling” That is a thorough process to ensure that every source of noise is excluded.

• The manuscript provided for review is not showing the figures, tables in the right inserts, which made the review a bit of a harder job.

• “Consequently, for faster access to be achieved, efficiency gains must be realised through alternative approaches.” The approaches suggested from here down are not technological alternatives to NLP, rather they are practical approaches to solve the studied problem. As a reader, I would expect that the focus throughout the manuscript to be on finding a solution for the streamlining problem rather than to find a specific technological solution.

• “the methodological framework developed here is able to be applied to other areas where identification of text and semantic similarity is needed.” If the methodological framework did not prove itself in the situation in hand, how is it expected to be taken forward for other area of application?

• “Additionally, a retrospective review of HTA reports from different jurisdictions may uncover substantial semantic overlap” Could you provide the reader with a justification for this assumption?

• “The more mature the field is, the more useful our method may be, since agreed terminology may recur more often.” What are the manuscript’s criteria to define ‘field maturity’?

• “NLP to compare texts for semantic similarity, remembering that these methods are applicable well beyond HTA and could even play a key role in benchmarking LLMs.” I am sure that you have reached this conclusion with elaborate efforts, but it needs to be more convincingly clear to the reader.

Thanks again. In this review, the focus is on the reader understanding and expanding the target audience of the manuscript. The questions are genuinely based on the reader trying to understand the text and connecting the dots in the information conveyed in it.

I appreciate the research efforts and all the work put into the study and I repeat my gratitude to be given this opportunity.

**********

what does this mean?). If published, this will include your full peer review and any attached files.

**Do you want your identity to be public for this peer review?**  If you choose “no”, your identity will remain anonymous but your review may still be made public.

For information about this choice, including consent withdrawal, please see our Privacy Policy

Reviewer #1: No

Reviewer #2: **Yes:**  Yasser Abdullah

**********

**Figure resubmission:**

**Reproducibility:**

To enhance the reproducibility of your results, we recommend that authors of applicable studies deposit laboratory protocols in protocols.io, where a protocol can be assigned its own identifier (DOI) such that it can be cited independently in the future. Additionally, PLOS ONE offers an option to publish peer-reviewed clinical study protocols. Read more information on sharing protocols at https://plos.org/protocols?utm_medium=editorial-email&utm_source=authorletters&utm_campaign=protocols

# Author comment: Author response to Decision Letter 1

**DOI:** 10.1371/journal.pdig.0001657.r002

21 May 2026

Attachment Submitted filename: Response to Reviewers.docx

# Editor report: Decision Letter 1

**DOI:** 10.1371/journal.pdig.0001657.r003

**Authors:** Dukyong Yoon

26 Jul 2026

A Natural Language Processing Approach to Determine Whether Streamlined Health Technology Assessment for Multi-Indication Medicines is Feasible

PDIG-D-26-00248R1

Dear Mr Luc,

We are pleased to inform you that your manuscript 'A Natural Language Processing Approach to Determine Whether Streamlined Health Technology Assessment for Multi-Indication Medicines is Feasible' has been provisionally accepted for publication in PLOS Digital Health.

Before your manuscript can be formally accepted you will need to complete some formatting changes, which you will receive in a follow-up email from a member of our team.

Please note that your manuscript will not be scheduled for publication until you have made the required changes, so a swift response is appreciated.

IMPORTANT: The editorial review process is now complete. PLOS will only permit corrections to spelling, formatting or significant scientific errors from this point onwards. Requests for major changes, or any which affect the scientific understanding of your work, will cause delays to the publication date of your manuscript.

If your institution or institutions have a press office, please notify them about your upcoming paper to help maximize its impact. If they'll be preparing press materials, please inform our press team as soon as possible -- no later than 48 hours after receiving the formal acceptance. Your manuscript will remain under strict press embargo until 2 pm Eastern Time on the date of publication. For more information, please contact digitalhealth@plos.org.

Thank you again for supporting Open Access publishing; we are looking forward to publishing your work in PLOS Digital Health.

Best regards,

Dukyong Yoon

Section Editor

PLOS Digital Health

***********************************************************

**Additional Editor Comments (if provided):**

**Reviewer Comments (if any, and for reference):**
